# Biochar induced improvement in root system architecture enhances nutrient assimilation by cotton plant seedlings

**DOI:** 10.1186/s12870-021-03026-1

**Published:** 2021-06-11

**Authors:** Lei Feng, Wanli Xu, Guangmu Tang, Meiying Gu, Zengchao Geng

**Affiliations:** 1 College of Natural Resources and Environment, Northwest Key Laboratory of Plant Nutrition and Agro-Environment, Ministry of Agriculture, Northwest A & F University, Yangling, 712100 China; 2 Xinjiang Academy of Agricultural Sciences Institute of Soil Fertilizer and Water Conservation, Urumqi, 830092 China; 3 Xinxiang Academy of Agricultural Sciences Institute of Microbial Application, Urumqi, 830091 China

**Keywords:** Gdh2,3, Nitrogen assimilation enzyme, Cytokinin, Fine root, Biochar

## Abstract

**Background:**

Raising nitrogen use efficiency of crops by improving root system architecture is highly essential not only to reduce costs of agricultural production but also to mitigate climate change. The physiological mechanisms of how biochar affects nitrogen assimilation by crop seedlings have not been well elucidated.

**Results:**

Here, we report changes in root system architecture, activities of the key enzymes involved in nitrogen assimilation, and cytokinin (CTK) at the seedling stage of cotton with reduced urea usage and biochar application at different soil layers (0–10 cm and 10–20 cm). Active root absorption area, fresh weight, and nitrogen agronomic efficiency increased significantly when urea usage was reduced by 25% and biochar was applied in the surface soil layer. Glutamine oxoglutarate amino transferase (GOGAT) activity was closely related to the application depth of urea/biochar, and it increased when urea/biochar was applied in the 0–10 cm layer. Glutamic-pyruvic transaminase activity (GPT) increased significantly as well. Nitrate reductase (NR) activity was stimulated by CTK in the very fine roots but inhibited in the fine roots. In addition, *AMT1;1, gdh3*, and *gdh2* were significantly up-regulated in the very fine roots when urea usage was reduced by 25% and biochar was applied.

**Conclusion:**

Nitrogen assimilation efficiency was significantly affected when urea usage was reduced by 25% and biochar was applied in the surface soil layer at the seedling stage of cotton. The co-expression of *gdh3* and *gdh2* in the fine roots increased nitrogen agronomic efficiency*.* The synergistic expression of the ammonium transporter gene and *gdh3* suggests that biochar may be beneficial to amino acid metabolism.

**Supplementary Information:**

The online version contains supplementary material available at 10.1186/s12870-021-03026-1.

## Background

Biochar is produced via dry carbonization, pyrolysis, or gasification of biomass, whereas hydrochar is produced as slurry in water by hydrothermal carbonization of biomass under press. Biochar is fine-grained, porous, of high specific surface area, and rich in functional groups and nutrients such as nitrogen, phosphorus, and potassium [1–3]. Biochar application has been found to be an effective, environmentally-friendly agricultural management technology for improving nitrogen utilization efficiency while reducing nitrogen fertilizer application. It not only improves nitrogen absorption but also enhances nitrogen assimilation by regulating root morphology and related physiological and metabolic processes [4–6]. Biochar has been proposed as a soil amendment to enhance nutrient retention, reduce nutrient losses, improve soil fertility and crop growth, and sequestrate carbon [7, 8]. In some cropping systems, biochar addition helps to reduce nitrogen fertiliser input while maintaining productivity [9] as biochar serves as a good complement to site preparation techniques that are frequently used for capturing nitrogen from nitrate so as to increase rhizosphere nitrogen bioavailability in alkaline soils [10]. According to other reports, biochar application level influences soil nutrient and plant root phenotype [11, 12]. Moreover, studies suggest that biochar changes crop nitrogen utilization efficiency and increases rhizosphere microbial community diversity [13, 14] which is strongly associated with root order [15]. Biochar increases microbial biomass [16] and especially the abundance of ammonia-oxidizing bacteria (AOB) which are closely related to the nitrogen cycle [17] and cause great variation in the NO_3_^−^-N/NH_4_^+^-N ratio in the rhizosphere of different root orders. Though there are many studies on the effects of AOB on the NO_3_^−^-N/NH_4_^+^-N ratio, little is known about the mutual effects between mineral nitrogen, rhizosphere AOB community, and root order when biochar is applied in gray desert soils.

The biochar-induced changes in the interaction between mineral nitrogen and hormones may influence root development along with nitrogen assimilation [18, 19]. For instance, biochar addition alters root morphology (larger specific root length, smaller root diameter, and lower root tissue mass density) to facilitate nitrogen uptake, indicating good proliferation of the roots regardless of the fertilization level [20, 21]. Di Lonardo et al. [22] found that biochar decreased the ethylene concentration and increased the number of roots of tissue-cultured poplar, indicating that root phenotype is significantly affected by biochar. It is suggested that biochar changes during root development, which may contribute to the nitrogen cycle and particularly help to increase the opportunity of capturing nitrogen from fertilizer and soil [23, 24]. However, there is still insufficient evidence supporting that biochar influences nitrogen metabolism via inducing root phenotypic changes.

Additionally, more key links may exist between biochar and the interaction of nitrogen metabolism and gene expression of root phenotype [25–27], and the effect of biochar on the physiological activities (nitrogen availability and auxin changes) of fine roots is found to be more significant [28, 29]. Recent research reported that 50 and 100 g kg^−1^ of biochar had the same effect in improving nitrogen metabolism, indicating that such effect of biochar may be dose-independent but hormone activation-associated [30]. An investigation demonstrated that biochar addition improved nitrogen assimilation by boosting the activities of glutamate dehydrogenase (GDH), glutamine synthetase (GS), glutamine oxoglutarate aminotransferase (GOGAT), and nitrate reductase (NR) [31]. Hashem et al. [32] revealed that biochar enhanced the nitrogen assimilation efficiency of chickpeas by increasing the NR activity. An in-depth examination indicates that the DOMs of biochar promote nitrogen assimilation and improve nitrogen efficiency by stimulating NR and GS gene expression [33]. Though there are large numbers of reports on enzymes in various plant organs and crop species, the mechanism of nitrogen metabolism regulation by biochar-induced changes in root phenotype remains to be unraveled.

Biochar influences the level and spatial distribution pattern of hormones, which may be the major factor interfering with the activities of the key enzymes involved in nitrogen metabolism [34, 35]. Waqas et al. [36] believed that changes in jasmonic acid signal in their study reflected an alleviating effect of biochar on biotic stress. Recently, studies show that biochar stimulates the gibberellins pathway and promotes the growth of tomato and wheat plants [37, 38]. Conversely, Hale et al. [39] found that 600 °C-pyrolysed pine sawdust biochar had no effect on auxin synthesis. Earlier research emphasizes that the balance between root function and root growth depends on the ethylene signaling pathway and enhanced H_2_O_2_ accumulation [40]. Although biochar-independent changes in plant endogenous hormones occur, it is unclear whether biochar can interfere with CTK metabolism and further affect the GDH and ammonium transporter (AMT) coding genes.

Some studies showed that GDH was not detected in each organ compared with the wild type with the gdh1-2–3 mutant. Root GDH activity reduction of 25% was only achieved in the gdh2 mutant, while activity increase of 30% in the root system was achieved in the gdh3 mutant [41]. Sun et al. [25] found that the nitrogen metabolism-related omics characteristics and transcription levels (zmGS1and zmAS1) in maize were significantly up-regulated when nitrogen fertilizer was reduced and biochar was applied. Transcriptomic analysis of tomato by Jaiswal et al. [27] demonstrated that biochar has a priming effect on gene expression; the group also found that the up-regulated genes are associated with plant growth such as jasmonic acid, CTK, auxin, and cell wall. Kumar et al. demonstrated that AMT1 affects not only the interaction between NH_4_^+^-N and NO_3_^−^-N during lateral root growth but also that between auxin and NH_4_^+^in rice roots [42]. Some studies showed that biochar regulates plant genes by interfering with microbial signals under abiotic stress [43, 44]. Thus, biochar may affect the synergistic effect of AMT and GDH genes by multiple ways. However, the mechanism of biochar-mediated root nitrogen metabolism has not yet been elucidated. Although it has been widely reported that there are significant differences in nutrient uptake, nitrogen storage, and phenotype between root orders [45, 46], there are few studies on nitrogen metabolism or nitrogen assimilation of different root orders under reduction of nitrogen application, which makes it impossible for us to fully understand the mechanism of nitrogen metabolism under the condition of nitrogen reduction with biochar addition.

The objectives of this study were to evaluate the changes in soil parameters (NH_4_^+^, NO_3_^−^, and AOB abundance), nitrogen-metabolizing enzymes in cotton roots (NR, GDH, glutamic-pyruvic transaminase (GPT), and GS), and cotton root biological characters (root active absorbing area, biomass, and nitrogen agronomic efficiency) caused by biochar addition with the reduction of urea. Furthermore, the effects of cotton AMT and *gdh* on the biochar-induced nitrogen metabolism were investigated so as to learn the consistence and difference between the expressions of *AMT* and *gdh* under the ammonium assimilation pathway and how CTK-stimulated nitrate metabolism contributes to seedling growth. Three hypotheses were tested: (i) biochar addition would alter soil biochemical properties, have a positive effect on the soil nitrogen status, and increase the AOB abundance; (ii) the biochar-induced effects would favor the root intraspecific variation-caused crosstalk between *AMT1;1* and *gdh2*; and (iii) seedlings in the treatments with biochar application and reduced urea usage would show a higher nitrogen metabolism and growth rate.

## Materials and methods

### Plant material

Seeds of *Gossypium hirsutum* 49, purchased from Xinjiang Jiu He Seeds Co. Ltd., Xinjiang Uygur Autonomous Region, China, were surface sterilized for 20 min in sterile water and placed on Petri dishes. After 48 to 72 h of *dormancy release* at 4 °C in the dark, the plates were transferred to a growth pot in a greenhouse for germination.

### Experimental design

The plastic pots (21 cm in height and 20 cm in diameter) used in this trial each contained 7.5 kg gray desert soil. The following treatments were set up:

- ck: neither urea nor biochar was applied;
- sb: 3.76 g urea kg^−1^ soil applied in the 0–10 cm soil layer;
- bb: 3.76 g urea kg^−1^ soil applied in the10–20 cm soil layer;
- soa: 25% less urea as compared with sb plus 37.28 g biochar kg^−1^ soil applied in the 0–10 cm soil layer;
- sob: 50% less urea as compared with sb plus 37.28 g biochar kg^−1^ soil applied in the 0–10 cm soil layer;
- boa: 25% less urea as compared with bb plus 37.28 g biochar kg^−1^ soil applied in the10–20 cm soil layer;
- bob: 50% less urea as compared with bb plus 37.28 g biochar kg^−1^ soil applied in the10–20 cm soil layer;

The properties of the soil and biochar obtained by pyrolysis of cotton straw at 450 °C used are shown in Table 1. Twelve healthy cotton seedlings were transferred to each pot and then thinned to five after establishment.

**Table 1:** Chemical properties of the soil and the biochar used in this study

Material	Organic matter/g kg^−1^	Available nitrogen/mg kg^−1^	Available phosphorus/mg kg^−1^	Available potassium/ mg kg^−1^	Total salt/g kg^−1^	pH	Soil electrical conductivity/mS cm^−1^
Soil	8.54	54 .33	43.12	102.45	11.02	8.6	0.33
Biochar (Bc)	406.33	36.57	918.74	12.30	60.97	10.5	3.70

The pots were randomly placed in a nursery in Xinjiang Agricultural University (E 43°49′07″, N 81°51′16″). In the meanwhile, soil moisture content is maintained at 60% ~ 65% of field water capacity, 14 h of light and 10 h of darkness, the temperature is maintained at 15 °C to 30 °C. After 20 days of growth, the cotton plants were removed from the pots and separated into above- and below-ground fractions. After rhizosphere soils were collected, the roots were carefully washed under running water and further divided into fines roots (*d* = 0.1–2 mm) and very fine roots (*d* < 0.1 mm). Segments (20 mm in length from the apex) of both the fine and very fine roots were stored in liquid nitrogen before high-throughput sequencing.

### Enzyme activity analysis

For crude enzyme extraction, root samples were pretreated with 2 mL imidazole in a cold mortar over ice and ground after adding 0. 05 mol/L HCl (pH 7.2). The homogenate was left to stand for 30 min, filtered through two layers of gauze, and centrifuged at 12,000 g for 20 min. All the above operations were carried out at 4 °C.

The reaction mixture for GOGAT activity determination consisted of 0.4 mL 20 mmol/L L-glutamine, 0.5 mL 20 mmol/L α-ketoglutaric acid, 0.1 mL 10 mmol/L KCl, 0.2 mL 3 mmol/L NADH, 0.3 mL crude enzyme solution, and 1.5 mL 25 mmol/L tris–HCl buffer (pH = 7.6). After the reaction was initiated, enzyme activity was measured continuously for a time period during which one extinction value at 340 nm was recorded every 20 s with a photometer until the optical density showed a steady sevenfold decrease. GOGAT activity was presented as the produced amount of the reduction product of NADH per unit of reaction time.

Assay of NR activity was carried out according to Cervilla et al. [47]. The reaction mixture in a final volume of 0.8 mL consisted of 0.5 mL 100 mmol/L potassium-phosphate buffer (pH 7.5), 0.1 mL 100 mmol/L KNO_3_, 0.1 mL 2 mmol/L NADH, and 0.1 mL of the crude enzyme solution. After incubation at 25 °C for 20 min, the reaction was terminated with 0.05 mL 1 mol/L zinc acetate. The reaction solution was centrifuged at 3000 g for 10 min, and 0.6 mL supernatant was diluted to 1 mL with distilled water. The nitrite formed was diazotized with sulfanilamide and reacted with N-(1- naphthyl) ethylene diamine dihydrochloride to produce azo dye which was measured spectrophotometrically at 540 nm. Glutamate dehydrogenase activity was also determined according to Cervilla et al.. Glutamic-pyruvic transaminase activity was measured according to Kasim and Dowidar and presented as the amount of pyruvate produced per unit time after 300-min reaction [48].

The results of the enzyme activities are presented as means ± standard errors (*n* = 5).

### Determination of cytokinin content

Cytokinin content was determined by enzyme linked immunosorbent assaysaccording to Yang [49]. Briefly, root samples were rinsed with cold tap water to remove adhesive soil particles, homogenized with a mortar and a pestle in liquid nitrogen, extracted with phosphate buffer (pH = 5) at -20 °C for 1 h, centrifuged at 10,000 × *g* and 4 °C for 15 min, incubated with horseradish peroxidase again at -20 °C for 30 min. Finally, cytokinin was quantified by enzyme label reader (Neogen 4700, USA).

### High-throughput sequencing

#### RNA extraction

Total RNA was extracted from the cotton root using TRIzol® Reagent (Ambion, USA) according to the manufacturer’s instructions and genomic DNA was removed using DNase I. Then, RNA quality and quantity were determined with a 2100 Bioanalyser (Agilent, USA) and a NanoDrop ND-2000 spectrophotometer (Thermo Fisher Scientific, USA), respectively. Only high-quality RNA sample (OD260/280 = 1.8 ~ 2.2, OD260/230 ≥ 2.0, RIN ≥ 6.5, 28S:18S ≥ 1.0, and > 1 μg) was used for preparation of the sequencing library.

#### Library preparation and Illumina sequencing

RNA-seq transcriptome library was prepared with the TruSeq™ RNA Sample Preparation Kit from Illumina (San Diego, CA) using 1 μg of total RNA. Briefly, messenger RNA was enriched with magnetic oligo (dT) beads and fragmented into short segments with fragmentation buffer. Double-stranded cDNA was synthesized using the SuperScript double-stranded cDNA synthesis kit (Invitrogen, CA) with random primers. The synthesized cDNA was then purified, end repaired, phosphorylated, and polyA tailed. Libraries were size selected on 2% Low Range Ultra Agarose and PCR amplified using Phusion DNA polymerase (NEB). After quantification with TBS380, the paired-end RNA-seq library was sequenced with the Illumina HiSeq xten/NovaSeq 6000 sequencer (2 × 150 bp read length).

#### Read mapping

The raw reads were processed and quality controlled by SeqPrep (https://github.com/jstjohn/SeqPrep) and Sickle (https://github.com/najoshi/sickle) with default parameters to obtain high-quality clean reads. Then, clean reads were aligned to the reference genome using HISAT2[50]. The aligned reads were assembled using the reference-based assembler StringTie [51].

#### Differential expression analysis

To identify differentially expressed genes (DEGs) between two different samples, the expression level of each transcript was calculated based on transcripts per million reads (TPM). Gene abundances were quantified using RSEM software [52]. Essentially, differential expression analysis was performed using DESeq2/DEGseq/EdgeR with Q ≤ 0.05, and genes with |log_2_FC|> 1 and Q ≤ 0.05 (DESeq2 or EdgeR)/Q ≤ 0.001 (DEGseq) were considered DEGs [53].

### Determination of soil NO_3_^−^-N and NH_4_^+^-N

The collected rhizosphere soil samples where plant were grown for 20 days were extracted with 1 mmol/L KCl solution, and concentrations of NO_3_^−^ and NH_4_^+^ were colorimetrically measured with a CleverChem 380 random access analyser (Dechem-Tech, Hamburg, Germany) according to the manufacturer’s instructions [54].

### Ammonia-oxidizing bacteria

The rhizosphere soil samples were used for analysis of AOB. The qPCR reaction mixture consisted of 10 μL Quantitect SYBR green master mix (Qiagen, Valencia, CA, USA), 0.25 μL forward and reverse primers, 2 μL DNA template (~ 10–40 ng DNA), and nuclease-free H_2_O to a final volume of 20 μL. Standard curves (*R*^2^ > 0.99) were generated by amplifying using the serial dilutions of the synthesized copies of the target gene sequences. All qPCR reactions were performed in quintuplicate and amplification efficiencies ranged from 80 to 90.8%. Amplification specificity was determined using melt curve analysis. The 16 s rDNA fragments of AOB were amplified by nested PCR (nest-PCR) and the primer sequence was used. F27/R1492 is a common primer used for bacteria. CTO189F/CTO654R is a specific primer for AOB; F341/R518 is a V3 region specific primer for 16 s rDNA.

AOB primers CTO189F—5 'GCAGRAAAGYAGGGGATCG and CTO654R—5 'CTAGGYTTGTAGTTTCAAACGC were used [55].

### Statistical analysis

Genes with a Bonferroni *P* value < 0.05 were considered differentially expressed [56]. In addition to the paired t-test approach, the rank product method was used to detect differentially expressed genes according to the different levels of FDR. Transcripts satisfying both the above family-wise error rate level and FDR < 0.0001% were presented to achieve an optimal interpretation of the transcriptome. Genes were sorted by descending rank product values to provide a hierarchical list based on both strength and reproducibility, which was used as an input to identify groups of genes with the same or related annotated functions. BlastX was used to combine Unigene Sequence with NR (Non-redundant Protein Sequence Database in GenBank), Swiss-Prot (Swiss-Prot Protein Sequence Database), KEGG (Kyoto Encyclopedia of Genes and Genomes) and COG (Cluster ofOrthologous Groups of proteins were compared in the database (Evalue < 1E-5) to obtain the proteins with the highest sequence similarity to Unigene, thereby obtaining the protein functional annotation information of the Unigene. According to the NR annotation information, Blast2Go software was used for GO annotation. After the GO information of each Unigene was obtained, WEGO software was used for GO function classification statistic [57].

## Results

### Changes of NO_3_^−^-N and NH_4_^+^-N in the rhizosphere

There were clear differences in NO_3_^−^-N content between the rhizosphere of fine roots and that of very fine roots in the biochar application treatments (Table 2). In addition, there was strong interaction between biochar application amount and application depth. Rhizosphere NO_3_^−^-N content increased when biochar was applied regardless of the application depth. In the 0–10 cm layer, NO_3_^−^-N content was slightly higher in the rhizosphere of the very fine roots than in that of the fine roots. Rhizosphere NO_3_^−^-N content increased significantly when urea was reduced by 25% in the 0–10 cm layer. The content of NO_3_^−^-N in boam was 42% higher than that in bbm. In the 10–20 cm layer, the highest NO_3_^−^-N content in the rhizosphere of the very fine roots in bob was 6.33 mg L^−1^. This indicates that results of crop nutrient uptake and utilization based on fine roots (*d* < 2 mm) may not be sufficient. Results based on further divided root functional segments (*d* < 0.1 mm and 0.1 < *d* < 2 mm) may provide a clearer picture on the changes of nitrogen concentration in the rhizosphere.

**Table 2:** Soil mineral nitrogen (NO_3_^−^ and NH_4_^+^) in the rhizosphere of very fine (*d* < 0.1 mm) and fine (0.1 < *d* < 2 mm) roots of *Gossypium hirsutum* in the different treatments when urea/biochar was applied in the 0–10 or 1–20 cm soil layer

Soil layers	NO_3_^−^ concentration (mg/L)				NH_4_^+^ concentration (mg/L)			
	0	10 cm	10	20 cm	0	10 cm	10	20 cm
Treatment	< 0.1 mm	0.12 mm	< 0.1 mm	0.12 mm	< 0.1 mm	0.12 mm	< 0.1 mm	0.12 mm
ck	0.13 (0.04) d	0.21 (0.11) d	0.11 (0.01) d	0.42 (0.03) d	0.076 (0.021) bc	0.083 (0.009) b	0.084 (0.013) b	0.088 (0.014) b
s	7.33 (1.02) a	8.22 (0.58) a	3.46 (0.33) bc	4.62 (0.29) c	0.061 (0.005) c	0.069 (0.003) c	0.041 (0.009) c	0.042 (0.017) c
b	3.28 (0.31) c	3.75 (1.04) c	1.04 (0.19) cd	1.09 (0.38) d	0.089 (0.009) b	0.095 (0.013) ab	0.092 (0.016) ab	0.099 (0.006) ab
soa	7.65 (0.82) a	9.58 (0.54) a	4.12 (0.08) b	9.77 (0.36) a	0.104 (0.033) a	0.127 (0.012) a	0.107 (0.029) a	0.101 (0.024) ab
sob	5.62 (1.12) b	6.33 (0.24) b	3.09 (0.25) bc	4.73 (0.17) c	0.092 (0.005) ab	0.086 (0.015) b	0.085 (0.008) b	0.089 (0.019) b
boa	5.09 (0.49) bc	5.89 (0.78) b	3.01 (0.37) bc	7.09 (0.28) b	0.048 (0.008) d	0.054 (0.002) c	0.117 (0.028) a	0.125 (0.003)a
bob	4.16 (0.08) bc	4.57 (0.04) bc	6.33 (0.19) a	9.69 (0.05) a	0.083 (0.021) b	0.076 (0.004) bc	0.104 (0.002) a	0.118 (0.002)a

Note: The different lowercase letters indicate significant differences between treatments with a confidence interval at *P* < 0.05

There are seven treatments: ck (control), sb (conventional 3.76 g urea kg^−1^ soil applied in the 0–10 cm soil layer), bb (conventional nitrogen fertilization in the 10-20 cm soil layer), soa (urea was reduced by 25% on the basis of sb plus 37.28 g biochar kg^−1^ soil), sob (urea was reduced by 50% on the basis of sb plus 37.28 g biochar kg^−1^ soil), boa (urea was reduced by 25% on the basis of bb plus 37.28 g biochar kg^−1^ soil), and bob (urea was reduced by 50% on the basis of bb with 37.28 g biochar kg^−1^ soil). Results are presented as mean values for five plants with SE

Ammonium nitrogen showed a similar trend to that of NO_3_^−^-N in the rhizosphere. The content of NH_4_^+^-N in the rhizosphere of both the fine and very fine roots was significantly higher in the treatments with 25% urea reduction than in ck. In the 0–10 cm soil layer, NH_4_^+^-N decreased by 0.003 mg L^−1^ in the rhizosphere of the very fine roots while increased by 0.026 mg L^−1^ in the rhizosphere of the fine roots in the biochar treatments as compared with the 100% urea treatment. When biochar was applied in the 10–20 cm layer, NH_4_^+^-N decreased by 59% and 57% in boaf and boam, respectively (Table 2).

### Copy number of AOB

The copy number of AOB in the rhizosphere of the very fine and fine roots was 4.71–8.65 × 10^7^ and 4.53–10.8 × 10^7^, respectively (Table 3), which indicates that fine roots may have provided more ecological niches for AOB. The AOB copy number in the rhizosphere of very fine roots in boa was 5.44 × 10^7^, which was not much different from those in soa, sob, and bob and was slightly higher than that in ck. Compared with that in ck, the amount of AOB in the rhizosphere of fine roots in both layers decreased in the conventional urea application treatment, while that in the 25% and 50% urea reduction treatments (applied to the 0–10 cm layer) increased 1.86- and 1.78-fold, respectively. The amount of AOB in the rhizosphere of fine roots in boa was up to 1.08 × 10^8^.

**Table 3:** Copy numbers of ammonia-oxidizing bacteria (AOB) in the rhizosphere of very fine (*d* < 0.1 mm) and fine (0.1 < *d* < 2 mm) roots of *Gossypium hirsutum* in the different treatments

Treatment	Very fine root	Fine root
ck	4.71 × 10^7^	5.21 × 10^7^
s	8.65 × 10^7^	4.98 × 10^7^
b	6.86 × 10^7^	4.53 × 10^7^
soa	7.29 × 10^7^	9.70 × 10^7^
sob	6.58 × 10^7^	9.28 × 10^7^
boa	5.44 × 10^7^	1.08 × 10^8^
bob	7.83 × 10^7^	6.95 × 10^7^

The treatments are as follows: ck (control), sb (conventional 3.76 g urea kg^−1^ soil applied in the 0–10 cm soil layer), bb (conventional nitrogen fertilization in the 10–20 cm soil layer), soa (urea was reduced by 25% on the basis of sb plus 37.28 g biochar kg^−1^ soil), sob (urea was reduced by 50% on the basis of sb plus 37.28 g biochar kg^−1^ soil), boa (urea was reduced by 25% on the basis of bb plus 37.28 g biochar kg^−1^ soil), and bob (urea was reduced by 50% on the basis of bb with 37.28 g biochar kg^−1^ soil)

### The effects of urea reduction combined with biochar application on the key enzymes involved in nitrogen metabolism

**Fig. 1:**
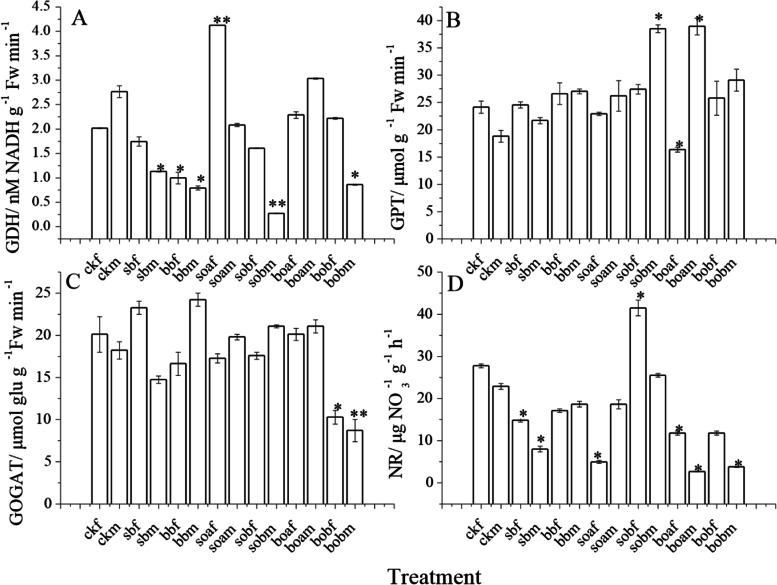
Activities of the key enzymes involved in nitrogen metabolism in the fine and very fine roots of *Gossypium hirsutum* in the different treatments. GDH = Glutamic dehydrogenase, GPT = Glutamic-pyruvic transaminase, GOGAT = Glutamic synthase, and NR = Nitrate Reductase. Fine roots are those with diameter between 0.1 and 2 mm, while very fine roots are those with diameter less than 0.1 mm. The treatments include ck (control), sb (conventional 3.76 g urea kg^−1^ soil applied in the 0–10 cm soil layer), bb (conventional 3.76 g urea kg^−1^ soil applied in the 10–20 cm soil layer), soa (urea was reduced by 25% on the basis of sb plus 37.28 g biochar kg^−1^ soil), sob (urea was reduced by 50% on the basis of sb plus 37.28 g biochar kg^−1^ soil), boa (urea was reduced by 25% on the basis of bb plus 37.28 g biochar kg^−1^ soil), and bob (urea was reduced by 50% on the basis of bb plus 37.28 g biochar kg^−1^ soil). The last letters f and m of each treatment indicate very fine root and fine root, respectively. Results are presented as mean values for five plants with SE. Asterisk indicates significant difference with a confidence interval at *P < 0.05 or **P < 0.01

Urea reduction combined with biochar application strongly interfered with the nitrogen metabolism in seedling roots, causing significant differences in the activities of the key enzymes involved in nitrogen metabolism. Compared with that in the conventional urea application treatment, the GDH activity in the very fine roots increased twofold in the 25% urea reduction treatment, while that in the fine roots decreased generally, with the largest decrease percentage of 91% in sobm. The GDH activity decreased significantly (*P* < 0.05) when urea application was reduced by 50% (Fig. 1A).

Compared with that in ck, GPT activity increased significantly (*P* < 0.05) in the fine roots in all fertilization treatments except in sob and boa. It decreased significantly (*P* < 0.05) in the very fine roots in boa. Reduced application of urea did not significantly influence GPT activity, indicating that 50% reduction in urea application in the 0–10 cm layer only influenced the GPT activity in fine roots while 25% reduction in urea application in the 10–20 cm layer influenced the GPT activity in both the fine and very fine roots (Fig. 1B).

The GOGAT activity showed a different changing trend from those of the above three enzymes in the biochar application treatments. When 50% less urea was applied in the 10–20 layer, GOGAT activity in both the very fine and fine roots decreased significantly (*P* < 0.05 and 0.01, respectively) as compared with ck (Fig. 1C). The NR activity increased significantly in sob but decreased in the other treatments as compared with ck and the conventional urea application treatment (Fig. 1D).

**Fig. 2:**
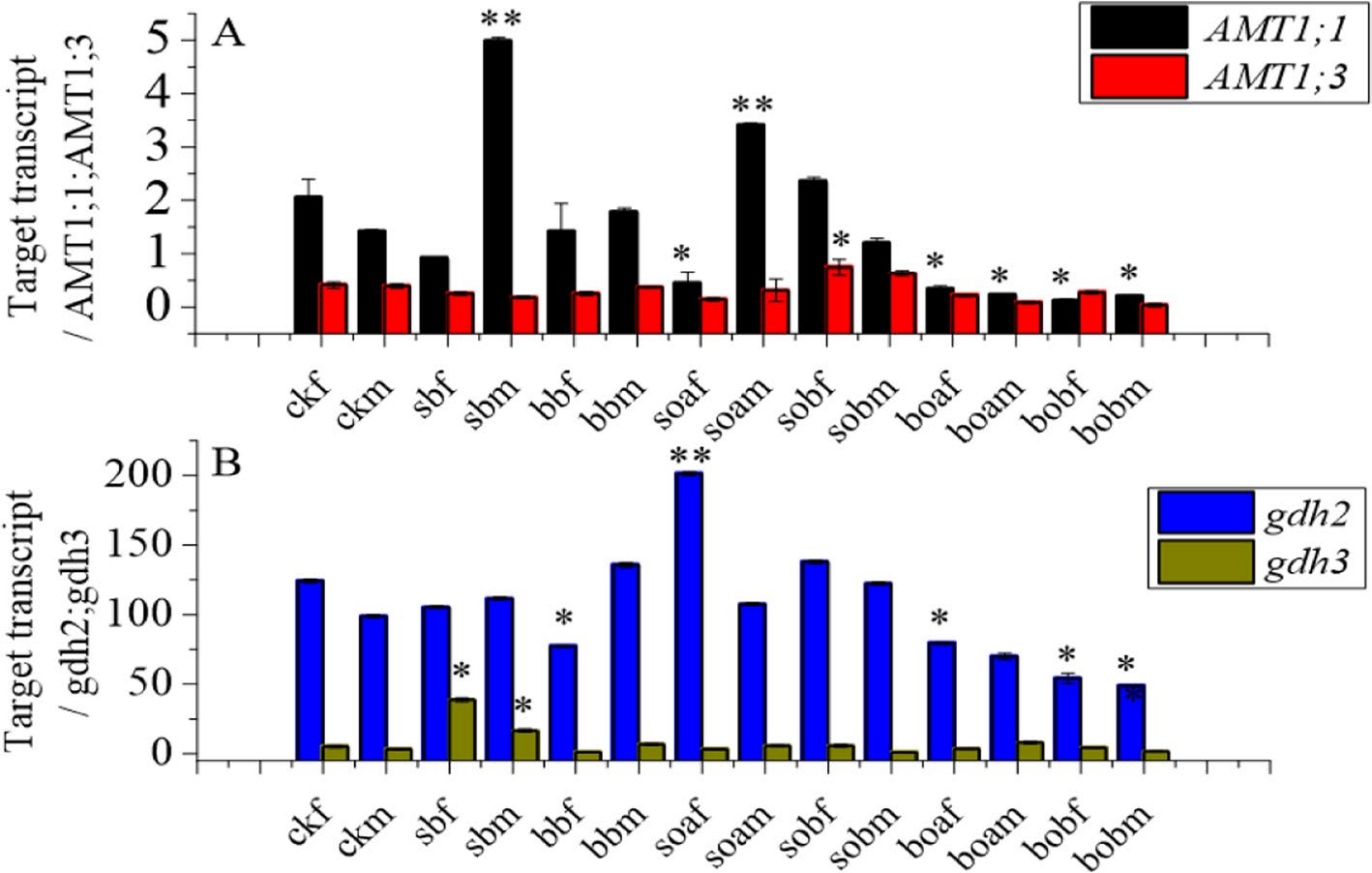
Expression levels of the *AMT1;1*, *AMT1;3*, *gdh2*, and *gdh3* genes in the very fine roots and fine roots of *Gossypium hirsutum* in the different treatments. Fine roots are those with diameter between 0.1 and 2 mm, while very fine roots are those with diameter less than 0.1 mm. The treatments include ck (control), sb (conventional 3.76 g urea kg^−1^ soil applied in the 0–10 cm soil layer), bb (conventional 3.76 g urea kg^−1^ soil applied in the 10–20 cm soil layer), soa (urea was reduced by 25% on the basis of sb plus 37.28 g biochar kg^−1^ soil), sob (urea was reduced by 50% on the basis of sb plus 37.28 g biochar kg^−1^ soil), boa (urea was reduced by 25% on the basis of bb plus 37.28 g biochar kg^−1^ soil), and bob (urea was reduced by 50% on the basis of bb plus 37.28 g biochar kg^−1^ soil). The last letters f and m of each treatment indicate very fine root and fine root, respectively. Results are presented as mean values for five plants with SE. Asterisk indicates significant difference with a confidence interval at *P < 0.05 or **P < 0.01

Previous studies have shown that AMTs play an important role in NH_4_^+^ absorption and transportation and directly influence nitrogen metabolism and root development. Significant changes in the transcription level of the AMTs occurred in the sample (Table S1). As shown in Fig. 2, both the *AMT1;1* and *AMT1;3* gene expression levels were up-regulated or down-regulated in the very fine and fine roots, and their changes became more prominent with larger urea reduction. The expression of *AMT1;1* was down-regulated 2.14-fold in soam but up-regulated 3.91-fold in soaf (Table 4). Biochar exhibited a stimulating effect on the express of AMT genes in roots when applied in the 0–10 cm layer but displayed an inhibiting effect when applied in the 10–20 cm layer (Fig. 2A). As another group of transcription factors, gdh significantly influenced nitrogen efficiency. The expression of *gdh2* in the very fine roots increased by 62% in soa, decreased significantly in bb, boa, and bob, but did not change much in sb and sob as compared with ck. The expression of gdh in the fine roots decreased significantly in bob but did not change much in the other treatments (Fig. 2B).

**Table 4:** Significantly altered-transcript abundances of *gdh* and AMT in the very fine (*d* < 0.1 mm) and fine (0.1 < *d* < 2 mm) roots of *Gossypium hirsutum* in the different treatments

sb	fine root	Down	Gohir.D12G215400	*gdh2*	-1.69	9.23E-10
			Gohir.D10G160800	*AMT1;1*	-2.32	2.40E-13
			Gohir.D10G160900	*AMT1;1*	-2.27	9.53E-13
			Gohir.A10G106700	*AMT1;1*	-4.10	4.13E-13
Treatment	Organ	Regulation	GG	Description	log 2FC	FDR
soa	very fine root	Up	Gohir.D03G104800	*gdh2*	1.06	1.76E-09
			Gohir.A10G106700	*AMT1;1*	3.91	3.26E-12
			Gohir.A08G213100	gdh3	3.44	1.13E-10
			Gohir.D03G104800	*gdh2*	1.30	4.44E-10
			Gohir.D08G230200	*gdh3*	5.00	5.82E-14
sob			Gohir.D03G104800	*gdh2*	1.55	1.28E-08
			Gohir.D12G198500	*gdh2*	1.06	1.61E-14
			Gohir.D08G230200	*gdh3*	1.78	3.02E-13
			Gohir.A10G106700	*AMT1;1*	2.60	9.13E-08
soa	very fine root	Down	Gohir.A12G212900	*gdh2*	-2.27	9.79E-06
			Gohir.A10G106400	*AMT1;1*	-1.18	1.16E-08
sob			Gohir.A10G106400	*AMT1;1*	-1.48	1.24E-12
			Gohir.A07G072500	*AMT1;2*	-3.97	4.40E-05
			Gohir.D07G077000	*AMT1;2*	-3.70	1.70E-05
sb	fine root	Up	Gohir.A08G213100	*gdh3*	1.25	6.46E-10
			Gohir.D08G230200	*gdh3*	1.90	5.96E-11
			Gohir.D11G140500	*AMT1;1*	1.45	7.45E-07
bb			Gohir.D10G160900	*AMT1;1*	2.08	5.55E-10
			Gohir.A11G134400	*AMT1;1*	1.73	2.47E-06
boa			Gohir.A08G213100	*gdh3*	2.24	3.24E-09
			Gohir.D08G230200	*gdh3*	2.67	1.93E-14
			Gohir.D10G160900	*AMT1;1*	2.40	3.63E-11
bb			Gohir.D02G050000	*AMT1;1*	-1.15	3.78E-12
			Gohir.A10G106400	*AMT1;1*	-2.87	1.40E-12
			Gohir.D10G160800	*AMT1;1*	-1.59	1.34E-13
			Gohir.A10G106700	*AMT1;1*	-1.38	2.78E-07
soa			Gohir.A10G106400	*AMT1;1*	-2.14	3.13E-14
sob			Gohir.A03G061200	*gdh2*	-0.80	8.43E-14
			Gohir.A02G044000	*AMT1;1*	-1.13	5.48E-15
			Gohir.A10G106400	*AMT1;1*	-6.61	5.50E-13
			Gohir.D10G160800	*AMT1;1*	-2.63	5.17E-13
			Gohir.A10G106700	*AMT1;1*	-4.48	2.77E-14
boa			Gohir.D11G140500	*AMT1;1*	-5.5	1.50E-05
			Gohir.D10G160800	*AMT1;1*	-1.86	2.76E-14
			Gohir.A10G106400	*AMT1;1*	-3.37	8.51E-13
			Gohir.A10G106700	*AMT1;1*	-8.35	6.18E-14

Arabidopsis and *pium hirsutum* genes identification

log fold change (log 2FC) between five replicated experiments. Transcripts exhibiting a FDR lower than 0.0001%. ck (control), sb (conventional 3.76 g urea kg^−1^ soil applied in the 0–10 cm soil layer), bb (conventional nitrogen fertilization in the 10–20 cm soil layer), soa (urea was reduced by 25% on the basis of sb plus 37.28 g biochar kg^−1^ soil), sob (urea was reduced by 50% on the basis of sb plus 37.28 g biochar kg^−1^ soil), boa (urea was reduced by 25% on the basis of bb plus 37.28 g biochar kg^−1^ soil), and bob (urea was reduced by 50% on the basis of bb plus 37.28 g biochar kg^−1^ soil). *AMT1;1* (Ammonium transporter1;1), *AMT1;2* (Ammonium transporter1;2) and gdh2(glutamate dehydrogenase 2)

### Crosstalk between CTK and NR

**Fig. 3:**
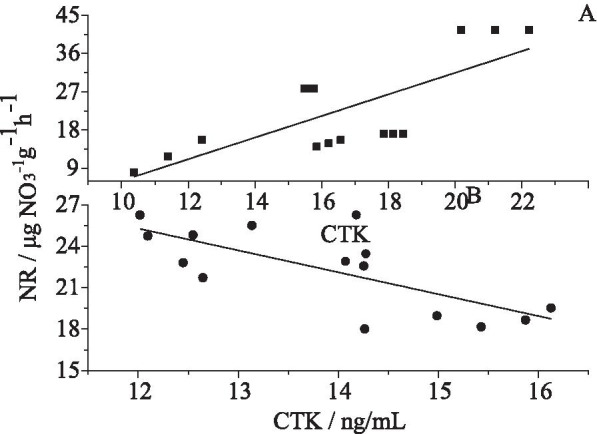
Relationship between CTK concentration and nitrate reductase activity in the very fine (**A**) and fine (**B**) roots of *Gossypium hirsutum.* Fine roots are those with diameter between 0.1 and 2 mm, while very fine roots are those with diameter less than 0.1 mm. The regression models are NR = 2.54 × CTK – 19.34 (*R*^2^ = 0.54, *P* < 0.001) and NR = 44.29 – 1.58 × CTK (*R*^2^ = 0.49, *P* < 0.002) for the very fine and fine roots, respectively

The activity of NR is strongly location-dependent and closely related to CTK. It was significantly stimulated in the very fine roots (ranging from 9 to 45) but inhibited in the fine roots (ranging from 16 to 27) by CTK (Fig. 3). The results indicate that biochar had enhanced the synergistic effect between CTK and NR. On the one hand, biochar may help to lower the metabolic cost in the fine roots, and on the other, it may speed up the nitrogen assimilation and turnover in the very fine roots.

### The effects of nitrogen reduction combined with biochar application on root traits

**Fig. 4:**
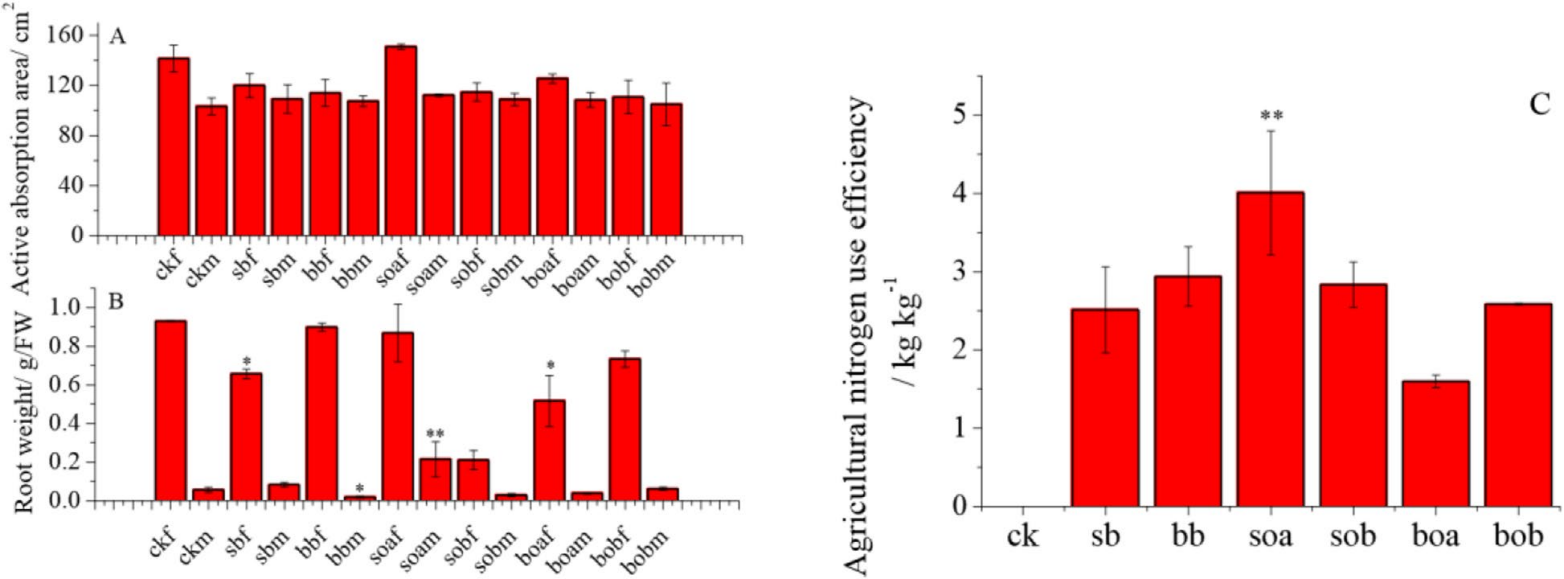
Active absorption areas and fresh weights of very fine and fine roots and seedling nitrogen agricultural efficiencies of *Gossypium hirsutum* in the different treatments. Fine roots are those with diameter between 0.1 and 2 mm, while very fine roots are those with diameter less than 0.1 mm. The treatments include ck (control), sb (conventional 3.76 g urea kg^−1^ soil applied in the 0-10 cm soil layer), bb (conventional 3.76 g urea kg^−1^ soil applied in the 10-20 cm soil layer), soa (urea was reduced by 25% on the basis of sb plus 37.28 g biochar kg^−1^ soil), sob (urea was reduced by 50% on the basis of sb plus 37.28 g biochar kg^−1^ soil), boa (urea was reduced by 25% on the basis of bb plus 37.28 g biochar kg^−1^ soil), and bob (urea was reduced by 50% on the basis of bb plus 37.28 g biochar kg^−1^ soil). The last letters “f” and “m” of each treatment indicate very fine roots and fine roots, respectively. Results are presented as mean values for five plants with SE. Asterisks indicate significant differences with a confidence interval at *P < 0.05 or **P < 0.01

The active absorption area of the very fine roots increased by 9.43 cm^2^, while that of the fine roots increased by 1.80–9.10 cm^2^ in soa as compared with the conventional urea application (Fig. 4A). The change in root fresh weight was similar to that of the active absorption area, that is, the fresh weight of the very fine roots decreased markedly while that of the fine roots decreased not much. The fresh weight of the very fine roots in sb and boa and that of the fine roots in bb decreased significantly (*P* < 0.05) as compared with ck. However, the fresh weight of the fine roots in soa increased significantly (*P* < 0.01) (Fig. 4B). The nitrogen fertilizer agronomic efficiency increased by 1.49 kg kg^−1^ in soa, decreased by 0.91 kg kg^−1^ in boa, and showed an increasing trend in the other treatments (Fig. 4C).

## Discussion

Improvement in the nitrogen use efficiency (NUE) of crops can not only reduce planting costs, but also reduce energy consumption related to chemical fertilizer production, which fundamentally alleviates global climate change [58, 59]. Combined application of biochar and chemical nitrogen fertilizer (e.g., urea) can effectively raise crop yield and NUE by slowing down nitrogen release, regulating microbial diversity, and stimulating nitrification while inhibiting denitrification, as compared with applying chemical fertilizer as the sole nitrogen source [60–62]. There are many studies focused on the increased nitrogen agronomic efficiency resulted from the improved soil nitrogen availability with biochar application, but there are few reports on molecular signals or root phenotypes related to nitrogen availability increase in rhizosphere [63, 64]. A recent report pointed out that root phenotype and architecture are influenced by soil biophysical and chemical properties such as mineral nitrogen, moisture, and temperature. Therefore, plant roots are of tremendous phenotypic plasticity in their cellular structure, anatomy, cell types, shapes, metabolisms, and biochemical profiles [15], causing big functional differences between root orders. Biochar application may enlarge such differences. For instance, biochar showed stronger effects on the morphological characteristics of roots with d < 0.5 mm [29]. Our results show that 25% urea reduction combined with biochar application led to nitrogen agronomic efficiency increase; in addition, AOB and mineral nitrogen increased in both the rhizospheres of the very fine and fine roots, with larger increases for the very fine roots. However, some studies found that the diversity of AOB decreased or did not change with biochar application, which is probably related to the changes in soil pH and NH_4_^+^ content [65]. The results suggest from genetic to phenotypic changes**.**

Extensive studies have shown that exogenous NH_4_^+^ triggers multiple specific changes in gene expression, metabolism, hormonal signaling, and root phenotype in rice and Arabidopsis [66, 67]*.* Therefore, the fact that improving the affinity of crop roots to NH_4_^+^ and NO_3_^−^ can raise nitrogen utilization efficiency may be closely related to the regulation of the key enzymes involved in nitrogen metabolism [42]. Generally, the activities of NR, GDH, and GS decrease with the decrease of nitrogen application level. This is because nitrogen levels in the metabolic and non-metabolic pools are decided by the exogenous mineral nitrogen concentration. In contrast, GOGAT is associated with crop nitrogen phenotype, that is, its activity can still be high under low nitrogen levels [5]. For instance, studies show that intraspecific root variation and increase in rhizosphere nitrogen availability improved nitrogen efficiency and promoted crop growth [68]. The activity variation trends of the key enzymes involved in nitrogen metabolism observed in this study are in accordance with those reported in the literature. We believe that AOB increase in the rhizosphere of the fine roots is an important cause for the changes in the enzyme activities. Biochar application helps to lower the energy consumption by higher plants in the synthesis of amino acids and nucleic acids, increases NH_4_^+^ transport into cells by AMT, and is favorable for root phenotype regulation by signaling molecules [69, 70].

AMTs in plants are encoded by the *AMT1* and *AMT2* subfamilies, and AMT1s is associated with efficient NH_4_^+^ transport in plants. *AMT1;1* in *Arabidopsis thaliana* is mainly expressed in the roots and leaves, while *AMT1;3* is only expressed in the roots; *both* are sensitive to nitrogen deficiency [71, 72]. When nitrogen is insufficient, *AMT1;1* and *AMT1;3, would be* significantly up-regulated in the root dermis. The up-regulation of *AMT1;2,* which is associated with carbon and nitrogen metabolism in roots [73], is much more striking. The results of this study showed that *AMT1;3* was significantly up-regulated in the very fine roots but obviously down-regulated in the fine roots. This suggests that *AMT1;3* in the fine roots was more readily regulated with biochar application. Structural variation in the cortex may be the main factor, and carbon variation in the rhizosphere may contribute to the nitrogen/carbon metabolism in roots to some degree.

Glutamate dehydrogenase (GDH) is a rate-limiting enzyme in ammonium assimilation when plants are under biotic or abiotic stresses. Metabolic processes involving NADH-gdh mainly occur in the roots [74]. In the dark or under stresses, a reversible reaction will occur to provide a carbon framework for the tricarboxylic acid cycle [75], which indicates that GDH is an intermediate of carbon/nitrogen metabolism and is closely related to the environment. In the urea reduction treatments, the GDH activity in the very fine roots increased significantly but that in the fine roots did not change much. The reason may be that the darker and more alkaline (pH 9.8) environment in these treatments had stimulated GDH protein coding and improved GDH activity [76]. In addition, biochar enhances seedling photosynthesis without inhibiting carbon metabolism [77]. It increases the content of soil organic matter or micro molecule, compensating for the lack of carbon to some degree [78, 79].

Generally, GDH in higher plants has a weak affinity for NH_4_^+^, and therefore, NH_4_^+^ is absorbed mainly by the GS/GOGAT pathway. The results of this study also showed that urea reduction plus biochar application had a strong inhibiting effect on the GOGAT activity in the very fine roots but a mild effect on that in the fine roots. During the process of ammonium assimilation in the induced GS/GOGAT pathway, exogenous ammonium ions may not decrease but even increase (to some extent) because biochar increases the AOB diversity in the fine root rhizosphere, which leads to an increase in NO_3_^−^ [80]. The results partly confirm that biochar addition compensated for the reduced urea application to supply plant-needed nitrogen, maintained the stability of amino acids in the nitrogen metabolic pool, and eventually mitigated the effects of reduced urea application to the roots.

NR is another key enzyme in nitrogen metabolism in higher plants. It is strongly influenced by NO_3_^−^ and light and interacts with hormones [81, 82]. The results showed that NR activity increased by 83% in sobm, only increased slightly in sobf, and decreased clearly in the other treatments as compared with ck. This suggests that the high concentration of NO_3_^−^ in the rhizosphere inhibited the growth of the very fine roots while stimulating the development of the fine roots. Consequently, NR activity was changed, which may be related to the fact that biochar can slow down root senescence [83]*.* The specific environment created by biochar is favorable for the expression of nitrate reductase/nitrite reductase in both the roots and shoots, which can improve plant/crop growth and yield [84]. In addition, a large number of experiments have confirmed that biochar significantly changes the rhizospheric environment [23, 24, 80]. In dark conditions, fine roots produce more CTK and consequently, NR activity increases; biochar application creates a darker environment and promotes nitrogen assimilation (Fig. 3A). Molecular dynamic analysis suggests a possible mechanism underlying the increased NR activity by CTK in the interface of biochar, which is that the small molecules induced by biochar may manage to access the active sites by lowering the energy barrier.

In short, when the urea dose was reduced by 25% and biochar was applied, *AMT1;1* and *gdh2* were up-regulated and ammonium assimilation was improved, which was closely related to the crosstalk between AMT and *gdh3*. Furthermore, the increased NR activity under the stimulation of CTK in the very fine roots also made an important contribution to ammonium assimilation.

## Conclusions

The presented results indicate that the crosstalk between the *gdh2* and *AMT1;1* signaling pathways influenced the ammonia assimilation of *Gossypium hirsutum* under the conditions of reduced urea plus biochar application. The nitrogen agronomic efficiency at the seedling stage was 1.51–3.99 kg kg^−1^ higher in the biochar application treatments than in ck. It is speculated that such difference was the direct result of *gdh2* upregulation and gdh3 supplementation. Physiologically, the increased GPT and GDH activities were the reason for the higher nitrogen assimilation at the seedling stage under reduced urea plus biochar application conditions. Though high nitrogen assimilation relies heavily on the GDH pathway, it is influenced by the function and development stage of the root system as well.

We further found that CTK can not only activate but also inhibit NR activity under the biochar application condition, which is directly related to the root turnover rate, age, and function. In the biochar application treatments, CTK displayed a stimulating effect on NR activity in the very fine roots but an inhibiting effect in the fine roots. This indicates that the application depth of biochar has a strong effect on root physiology. Overall, the biochar-induced upregulation of *gdh3* expression can partially explain the improvement of nitrogen assimilation in the fine roots.

In this study, we demonstrate that the effect of reduced nitrogen usage plus biochar application on nitrogen assimilation efficiency is strongly associated with hormone activation. Knowledge on the differential functions of very fine and fine roots will provide support for effectively improving nitrogen assimilation efficiency. We still need to further explore the interaction between various hormones and nitrogen metabolizing enzymes, that is, to further reveal the mechanism underlying the deep connection between root phenotype and nitrogen metabolism under reduced nitrogen usage plus biochar application. In addition, the influence of biochar on the net nitrogen release rates of different root sequences and its complex relationship with rhizosphere microbial diversity should also be considered as a focus of future research.

## Supplementary Information

Additional file 1: **Table S1.** Genetic analysis of differences between groups base on DEGseq.

Additional file 2: **Figure S1.** Heat map showing the expression level of metabolic genes in Gossypium hirsutum. Note: Each column corresponds to a sample, with labels shown below the columns; each row corresponds to a gene, with labels shown on the right of the rows; color denotes standardized gene expression level; the row dendrogram shows hierarchical gene clustering, with closer branches indicating closer gene expression levels; the column dendrogram shows hierarchical sample clustering, with closer branches indicating more similar of the expression patterns of the genes in the samples, that is, more similar the changing trends of the gene expression levels.

## Data Availability

The data that support the results are included within the article and its additional files. Other relevant materials are available from the corresponding authors on reasonable request.

## Abbreviations

GDH: Glutamic dehydrogenase
GPT: Glutamic-pyruvic transaminase
GOGAT: Glutamic synthase
NR: Nitrate Reductase
AMTs: Ammonium transporters
AOB: Ammonia oxidizing bacteria
sbf: Very fine root under conventional 3.76 g nitrogen kg^−^^1^ soil fertilization in 0 ~ 10 cm soil layers
sbm: Fine root under conventional 3.76 g nitrogen kg^−^^1^ soil fertilization in 0 ~ 10 cm soil layers
bbf: Very fine root under conventional nitrogen fertilization in 10 ~ 20 cm soil layers
bbm: Fine root under conventional nitrogen fertilization in 10 ~ 20 cm soil layers
soaf: Very fine root under nitrogen was reduced by 25% on the basis of sb with 37.28 g biochar kg^−^^1^ soil
soam: Fine root under nitrogen was reduced by 25% on the basis of sb with 37.28 g biochar kg^−^^1^ soil
sobf: Very fine root under nitrogen was reduced by 50% on the basis of sb with 37.28 g biochar kg^−^^1^ soil
sobm: Fine root under nitrogen was reduced by 50% on the basis of sb with 37.28 g biochar kg^−^^1^ soil
boaf: Very fine root under nitrogen was reduced by 25% on the basis of bb with 37.28 g biochar kg^−^^1^ soil
boam: Fine root under nitrogen was reduced by 25% on the basis of bb with 37.28 g biochar kg^−^^1^ soil
bobf: Very fine root under nitrogen was reduced by 50% on the basis of bb with 37.28 g biochar kg^−^^1^ soil
bobm: Fine root under nitrogen was reduced by 50% on the basis of bb with 37.28 g biochar kg^−^^1^ soil
