# Supplementary material for: Biochar induced improvement in root system architecture enhances nutrient assimilation by cotton plant seedlings
Source: BMC Plant Biol. 2021 Jun 11;21:269. doi: 10.1186/s12870-021-03026-1 (PMC8194105; doi:10.1186/s12870-021-03026-1)
Supplement: Supplementary file 1 — Additional file 1: Table S1. Genetic analysis of differences between groups base on DEGseq. [file 12870_2021_3026_MOESM1_ESM.docx]

Table S1 Genetic analysis of differences between groups base on DEGseq. |log2FC| >=**3.322** &p-adjust<**0.001**

| **diff_group** | **total DEG** | **up** | **down** | **AMTs; gdh (up)** | **AMTs; gdh**  **(down)** |
| --- | --- | --- | --- | --- | --- |
| sbf vs soaf | 1034 | 514 | 520 | 10.1% | 10.3% |
| bobm vs bbf | 872 | 220 | 652 | 4.2% | 7.6% |
| sbm vs sobf | 1490 | 855 | 635 | 6.7% | 4.6% |
| sbm vs sobm | 2044 | 1119 | 925 | 9.3% | 6.8% |
| bobm vs soam | 2866 | 1875 | 991 | 18% | 7.3% |
| sbf vs boaf | 1673 | 698 | 975 | 7.1% | 9.8% |
| sbm vs bbm | 1118 | 434 | 684 | 4.4% | 6.1% |
| sbf vs bobf | 590 | 321 | 269 | 13.2% | 10.1% |
| bobm vs boam | 1080 | 400 | 680 | 5.9% | 9.3% |
