# Supplementary figures and images for: Biochar induced improvement in root system architecture enhances nutrient assimilation by cotton plant seedlings
Source: BMC Plant Biol. 2021 Jun 11;21:269. doi: 10.1186/s12870-021-03026-1 (PMC8194105; doi:10.1186/s12870-021-03026-1)

Heatmap

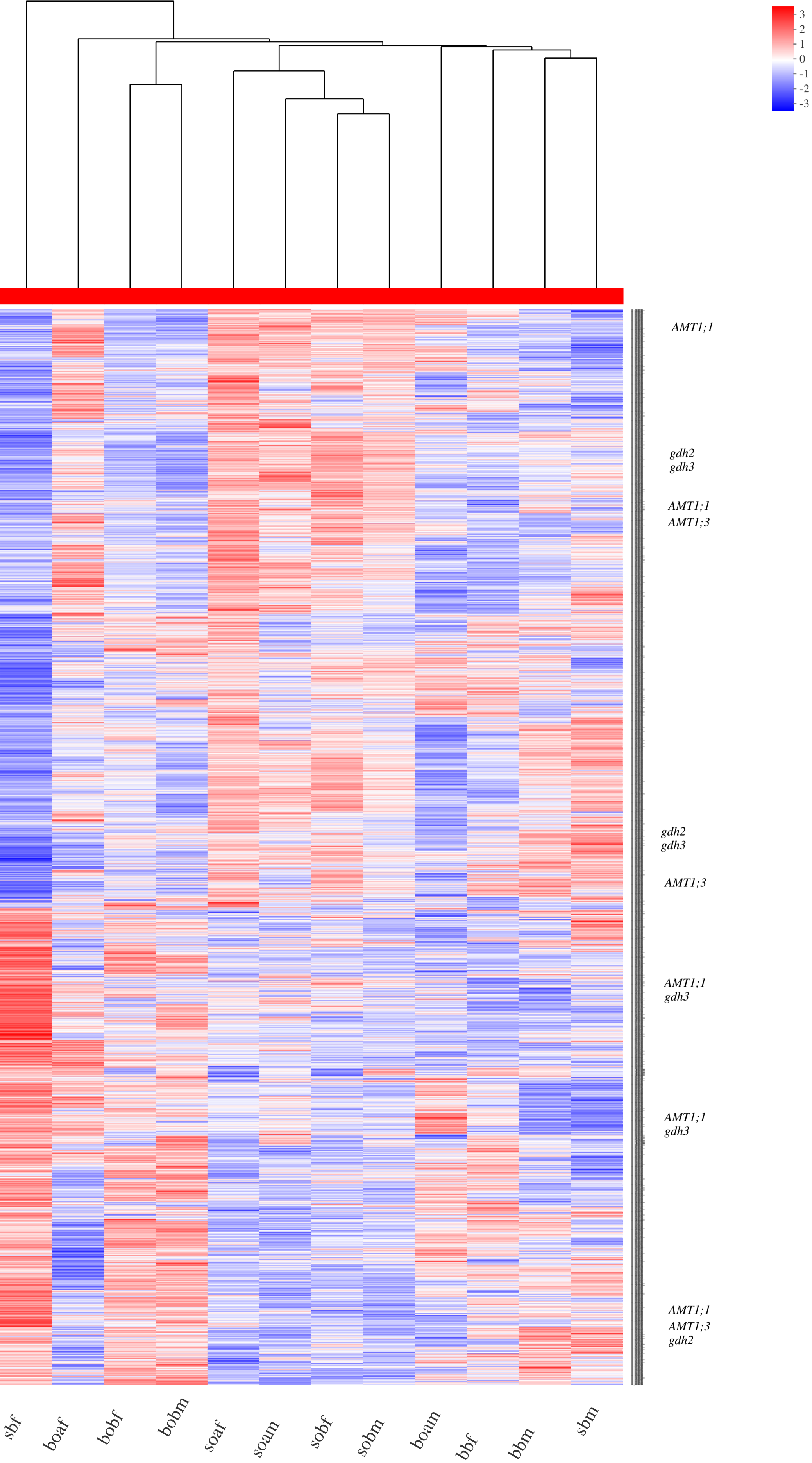

Supplement: Supplementary file 2 — Additional file 2: Figure S1. Heat map showing the expression level of metabolic genes in Gossypium hirsutum. Note: Each column corresponds to a sample, with labels shown below the columns; each row corresponds to a gene, with labels shown on the right of the rows; color denotes standardized gene expression level; the row dendrogram shows hierarchical gene clustering, with closer branches indicating closer gene expression levels; the column dendrogram shows hierarchical sample clustering, with closer branches indicating more similar of the expression patterns of the genes in the samples, that is, more similar the changing trends of the gene expression levels. [file 12870_2021_3026_MOESM2_ESM.pdf]
